# Predicting clinical response to costimulation blockade in autoimmunity

**DOI:** 10.1093/immadv/ltaa003

**Published:** 2020-11-25

**Authors:** Natalie M. Edner, Chun Jing Wang, Lina Petersone, Lucy S. K. Walker

**Affiliations:** Division of Infection and Immunity, Institute of Immunity and Transplantation, University College London, London, UK

**Keywords:** autoimmunity, type 1 diabetes, CD28 costimulation blockade, helper T cells, follicular helper T cells

## Abstract

Curbing unwanted T cell responses by costimulation blockade has been a recognised immunosuppressive strategy for the last 15 years. However, our understanding of how best to deploy this intervention is still evolving. A key challenge has been the heterogeneity in the clinical response to costimulation blockade, and an inability to predict which individuals are likely to benefit most. Here, we discuss our recent findings based on the use of costimulation blockade in people with type 1 diabetes (T1D) and place them in the context of the current literature. We discuss how profiling follicular helper T cells (Tfh) in pre-treatment blood samples may have value in predicting which individuals are likely to benefit from costimulation blockade drugs such as abatacept.

## Costimulation and autoimmunity

Autoimmune diseases arise as a consequence of damaging immune responses against self-tissues. The strong genetic associations between particular major histocompatibility antigens and susceptibility to autoimmune disease point to the importance of T cells in disease pathogenesis. T cell-directed therapies are therefore a promising strategy for the treatment of multiple autoimmune conditions. Costimulation blockade as an immunosuppressive strategy stems from the so-called ‘2 signal rule’ of T cell activation, which postulates that recognition of antigen via the T cell receptor alone is insufficient to trigger a T cell response; an additional ‘costimulatory’ signal delivered after binding of T cell-expressed CD28 to its ligands, CD80 (B7.1) or CD86 (B7.2), is required. It follows that inhibiting this second signal would therefore impair T cell immunity. Consistent with this, inhibiting the CD28 pathway by genetic deficiency or blockade ameliorates autoimmunity in multiple animal models [1], while augmenting CD28 signalling by transgenic expression of its ligands can promote autoimmunity, one example being the pancreatic islet expression of CD80 in mouse models of autoimmune diabetes [2].

## Costimulation blockade in type 1 diabetes

The natural inhibitor of CD28 is the immune regulator CTLA-4 that binds to the same ligands as CD28 but with higher affinity and, in the case of CD80, higher overall avidity. CTLA-4, therefore, acts as a competitive inhibitor, depriving CD28 of access to its ligands and limiting T cell costimulation. This function is augmented by the ability of CTLA-4 to physically remove its ligands from the surface of adjacent cells by a process of transendocytosis [3]. The ability of CTLA-4 to regulate CD28 signalling and T cell activation has led to the development of soluble CTLA-4 molecules for clinical use.

Abatacept (Orencia®) is a chimeric protein comprising the extracellular domain of human CTLA-4 fused to the Fc region of immunoglobulin (CTLA-4-Ig). It has proven clinically efficacious in the treatment of rheumatoid arthritis (RA) and was licensed by the FDA for use in this disease setting in 2005. In 2011, abatacept was trialled in the context of recent-onset type 1 diabetes (T1D) [4]. At the end of a 24-month-long randomised, double-blind, placebo-controlled study, individuals treated with abatacept displayed 59% higher serum C-peptide levels (indicative of insulin production) than those who received placebo. Furthermore, a subsequent follow-up study demonstrated that the beneficial effects of abatacept therapy were largely maintained even 1 year after cessation of treatment [5]. Despite the encouraging signs, heterogeneity in the response to abatacept treatment could also be observed. A clear understanding of which T cell populations are affected by costimulation blockade would help the identification of immune biomarkers and facilitate the selection of patients that would benefit from abatacept treatment.

## Which T cells are impacted by costimulation blockade?

Although CD28 costimulation is a key regulator of T cell activation, it is becoming increasingly clear that certain T cell populations are more dependent on this pathway than others. It has previously been shown that CD28 plays an important role in the development of follicular helper T cells (Tfh), the CXCR5-expressing T cell subset that collaborates with B cells to support humoral immunity. In fact, mice deficient in CD28 signalling (as a result of transgenic expression of CTLA-4-Ig [6], or germline deficiency of CD80 and CD86 [7]) were unable to generate Tfh and form germinal centres. Furthermore, T cells expressing less CD28 as a result of gene heterozygosity showed a reduced capacity for Tfh formation, despite normal proliferation [8]. These data suggest Tfh are prime candidates to be targeted by costimulation blockade.

**Figure 1:**
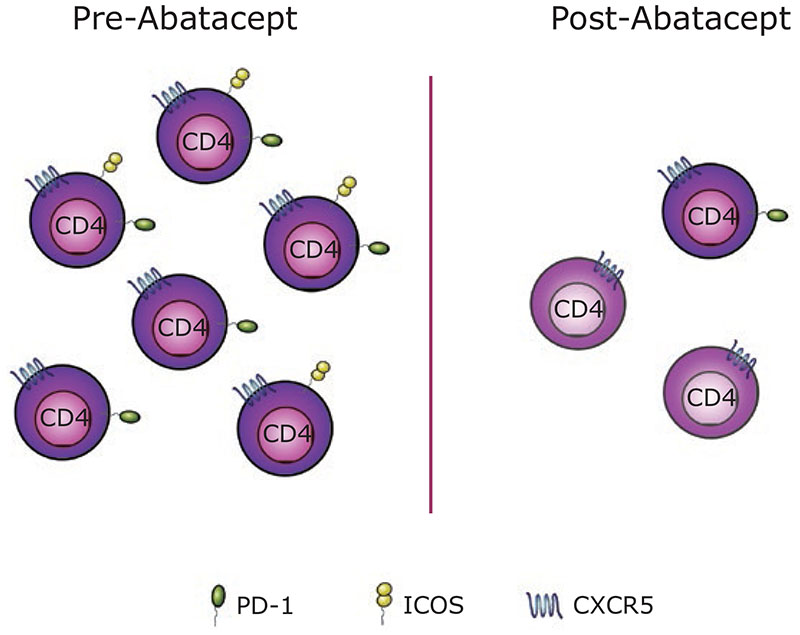
Abatacept treatment affects circulating Tfh cell frequency and their phenotype. Circulating CXCR5-expressing CD4 T cells (here called Tfh) can express PD-1, ICOS or both (left). Following abatacept treatment the frequency of circulating Tfh cells is reduced and the Tfh cells that remain show markedly lower expression of ICOS and, to a lesser extent, PD-1 (right).

We tested this idea using cryopreserved samples from recent-onset T1D patients, recruited in the 2011 clinical trial of abatacept [4]. CD4 T cells expressing CXCR5 can be found in the blood and are believed to contain memory Tfh that can home to germinal centres upon secondary challenge [9, 10]. Our analysis identified activated (ICOS-expressing) circulating Tfh cells to be one of the T cell populations most reduced by costimulation blockade [11, 12]. As well as a decrease in the number of circulating CXCR5+ T cells in response to abatacept, we also noted a profound change in their phenotype with a marked loss of PD-1 and a particularly significant reduction of ICOS expression (Fig. 1). The decrease in Tfh following costimulation blockade is consistent with studies conducted in other autoimmune diseases, including RA [13], multiple sclerosis [14], and Sjögren’s syndrome [15]. Previous work from Orban *et al*. suggested an effect of abatacept treatment on central memory T cells in T1D [16]. Since a substantial proportion of Tfh belongs to the central memory compartment [17], it is tempting to speculate that the reduction in central memory T cells reflects the loss of Tfh.

Interestingly, we identified another population of costimulation sensitive cells that expressed PD-1 and ICOS but lacked CXCR5 [11]. These cells resemble the peripheral helper T cells (Tph) recently described by Rao and colleagues that are believed to provide help to B cells at inflammatory sites (e.g. RA joint) [18]. Furthermore, in our study, regulatory T cells were also shown to be decreased by costimulation blockade as reported by others [16, 19].

A common characteristic among the T cell populations revealed to be abatacept-sensitive in our study was expression of the costimulatory receptor ICOS. This is in line with ICOS expression being highly CD28 driven and may explain why T cell populations dependent on ICOS signalling, such as Tfh, are particularly sensitive to CD28 costimulation blockade.

## Predicting clinical response to costimulation blockade

The heterogeneity in the clinical response to abatacept has generated a pressing need to develop predictive biomarkers. In RA, the presence of anti-citrullinated protein antibodies is known to be associated with a better response to abatacept [20, 21]. It has also been suggested that abatacept responders exhibit a more marked decrease in anti-citrullinated protein antibody levels after 12 months than non-responders [22]. A separate study of RA patients comparing the baseline serum proteome from five abatacept responders and five non-responders found cartilage oligomatrix protein to be significantly lower in serum from responders and suggested that combining this with measurement of fibronectin or lipopolysaccharide-binding protein could improve predictive power [23]. Attempts are underway to use serum proteomic analysis to develop prediction software that can guide the selection of diseasemodifying drugs in the RA setting [24].

In T1D, early findings showed that an increase in central memory T cell frequency was associated with a decline in C-peptide at the following measurement time point [16]. This change in central memory T cells was therefore proposed as an immunological marker for C-peptide loss that could inform how well an individual is responding to therapy. Two more recent studies used RNA sequencing to stratify abatacept responsiveness at the transcriptomic level. Data from Cabrera and colleagues indicated that a more proinflammatory profile at baseline was associated with a poorer response following abatacept treatment [25], while Linsley and colleagues showed that a higher B cell gene module expression 84 days after abatacept treatment initiation may predict C-peptide decline; however, this association was lost at later time points [26]. The latter study also highlighted the link between age at diagnosis and response to abatacept. Given the emerging evidence that individuals diagnosed before the age of 7 years exhibit distinct islet pathology and faster disease progression [27], it is likely that age at diagnosis will be a component of all approaches to predict sensitivity to immunotherapies in T1D.

**Figure 2:**
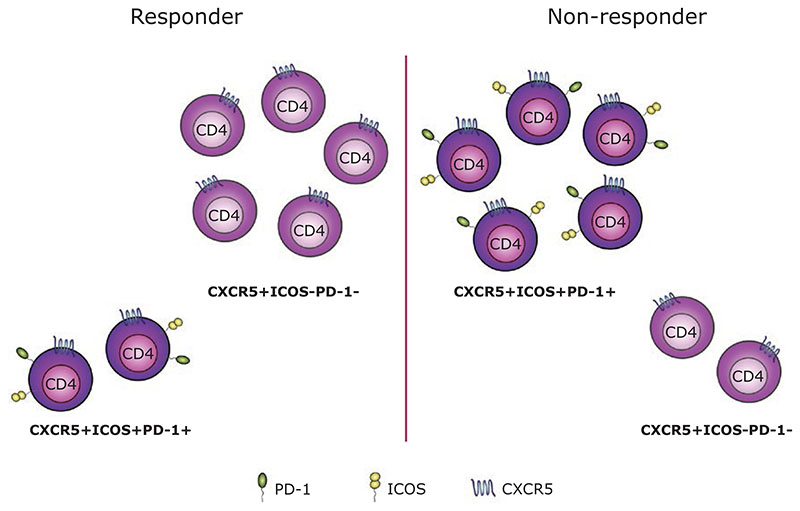
Baseline Tfh profile may help predict clinical response following abatacept treatment. Using a predictive modelling approach, we were able to show that the baseline Tfh profile may help predict an individual’s clinical response following abatacept treatment. Responders were shown to have a higher frequency of CXCR5^+^ICOS^-^PD-1^-^CD4^+^ T cells (left), while CXCR5^+^CD4^+^ T cells expressing both ICOS and PD-1 were more abundant in non-responders (right).

Our own analysis suggested an unanticipated link between baseline Tfh phenotypes and an individual’s subsequent clinical response to abatacept treatment [11]. By comparing profiles from responders and non-responders, we were able to build a predictive model using gradient boosting that could predict the response to abatacept with 85% accuracy and an AUC of 0.81. While the model drew on the combined frequencies of multiple populations, the population that held most predictive power was ICOS^+^ Tfh cells. Indeed, of the six populations that were most important for the generation of the prediction, five expressed CXCR5. Data-driven analysis using the CellCnn algorithm supported the link between Tfh phenotypes and sensitivity to abatacept treatment, with the expression of PD-1 and particularly ICOS on Tfh being associated with a poor clinical response (Fig. 2).

Since ICOS is highly CD28 dependent, it might be expected that individuals with strongly CD28-driven pathology would be characterised by high ICOS expression and show sensitivity to CD28 costimulation blockade. Notably, our data showed the exact opposite—individuals with high numbers of ICOS^+^ Tfh were less likely to respond to costimulation blockade. This observation could suggest that the baseline Tfh profile reflects an individual’s disease stage, with patients displaying higher frequencies of ICOS^+^ Tfh having more advanced disease and no longer being amenable to this intervention. Extrapolating this notion further, if Tfh profiles can indeed serve as a proxy for disease stage, they may be helpful in predicting the response to other interventions, not just costimulation blockade. However, these findings would first require corroboration in independent clinical trials involving larger numbers of participants.

## Concluding remarks

In summary, recent findings provide a further step forward in our understanding of how costimulation blockade perturbs the peripheral immune system and how to identify individuals who will benefit most from this treatment. An emerging theme is the sensitivity of B-helper T cell populations to costimulation blockade. One might speculate that T cell help for B cells, whether by Tfh or Tph, is a key CD28-mediated function, making these cells particularly effective readouts for costimulation blockade.

A better appreciation of the hierarchy of CD28 dependence among T cell subsets will help to shape our thinking on which disease settings may be amenable to costimulation blockade immunotherapy. In this regard, given the importance of tissue-resident T cells in autoimmune diseases, it will be of interest to determine the sensitivity of these cells to costimulation blockade. There is little data on this currently; however, in the context of influenza infection, it has been shown that at least some tissue-resident memory CD8 T cells in the lung are reliant on CD28 costimulation [28].

Regarding the use of biomarkers for patient stratification, it will be of interest to see whether predictive markers identified in abatacept-treated individuals are also applicable to recipients of next-generation CTLA4-Ig molecules and antagonistic anti-CD28 antibodies. Furthermore, it will be important to establish their utility across different disease settings and in recipients of combination therapy such as the abatacept and rituximab combination currently being trialled in T1D (NCT03929601).

## Data Availability

Data sharing is not applicable to this article as no new data were created or analysed in this study.
